# Soluble MICB protein levels and platelet counts during hepatitis B virus infection and response to hepatocellular carcinoma treatment

**DOI:** 10.1186/s12879-015-0754-x

**Published:** 2015-01-23

**Authors:** Hoang Van Tong, Le Huu Song, Nghiem Xuan Hoan, Bui Khac Cuong, Bui Tien Sy, Ho Anh Son, Do Quyet, Vu Quoc Binh, Peter G Kremsner, Claus Thomas Bock, Thirumalaisamy P Velavan, Nguyen Linh Toan

**Affiliations:** Vietnam Military Medical University, 160 Phung Hung Street, Ha Dong District, Ha Noi, Viet Nam; Tran Hung Dao Hospital, 108 Institute of Clinical Medical and Pharmaceutical Sciences, No 1 Tran Hung Dao Street, Hai Ba Trung District, Hanoi, Vietnam; Vietnam Military Medical Bureau, No 276 Nghi Tam, Tay Ho District, Hanoi, Vietnam; Robert Koch Institute, Seestrasse 10, D-13353 Berlin, Germany; Institute of Tropical Medicine, University of Tübingen, Wilhelmstr. 27, 72074 Tübingen, Germany; Fondation Congolaise pour la Recherche Medicale, P.O Box 2672, Brazzaville, Republic of Congo; Department of Pathophysiology, Vietnam Military Medical University, 160 Phung Hung, Ha Dong, Ha Noi, Vietnam

**Keywords:** Soluble MICB protein, Hepatitis B, Hepatocellular carcinoma, Cancer treatment, Platelet counts

## Abstract

**Background:**

The human major histocompatibility complex class I polypeptide-related sequence B (MICB) is a protein that modulates the NK and T cell activation through the NKG2D receptor and is related to several diseases including cancer.

**Methods:**

The study investigated the prognostic role of soluble MICB (sMICB) protein in the progression of HBV-related liver diseases and to HBV-related HCC treatment. The sMICB serum levels were measured in 266 chronic HBV-infected Vietnamese patients and in healthy controls, and correlated with clinical and laboratory parameters and with therapeutic interventions for HBV-related HCC.

**Results:**

Significant differences in both clinical and laboratory parameters were observed among the patient groups with different stages of hepatitis. The platelet counts were significantly decreased with disease progression (*P* < 0.001). The sMICB serum levels were significantly increased in HBV patients compared to healthy controls (*P* < 0.0001). Among the patients with different stages of hepatitis, asymptomatic individuals (ASYM) revealed higher sMICB serum levels while liver cirrhosis (LC) patients revealed lower sMICB serum levels (*P* < 0.0001) compared to other patient groups. Notably, the sMICB serum levels were decreased in treated HCC patient group compared to not-treated HCC patient group (*P* = 0.05). Additionally, the sMICB levels were significantly correlated with platelet counts in ASYM and HCC patients (r = −0.37, *P* = 0.009; and r = 0.22, *P* = 0.025, respectively).

**Conclusions:**

Our results demonstrate a potential role of sMICB serum levels and platelet counts during immune response to the HBV infection, liver disease progression and response to the HCC treatment.

## Background

Hepatitis caused by hepatitis B virus (HBV) is one of the major global health problems with an estimate of at least two billion people who had been infected with HBV. More than 240 million chronic HBV carriers and approximately 600,000 deaths were recorded annually either due to hepatitis B throughout the world [1]. HBV infection leads to a wide spectrum of pathologies including subclinical, acute self-limited (AHB) and fulminant hepatitis, an asymptomatic carrier state (ASYM), chronic hepatitis (CHB) progressing to liver cirrhosis (LC) and life threatening hepatocellular carcinoma (HCC). Approximately 50% of all HCC cases in childhood worldwide are caused by HBV infection [2]. HBV is highly prevalent in sub-Saharan Africa, Southeast Asia and parts of America with the infection rates ranging from 8% to 20%. In Southeast Asia, Vietnam has documented a prevalence of chronic hepatitis B up to 20% in the population, with approximately 10 million people living with chronic hepatitis B. In Vietnam, 23,300 deaths were registered in 2005, and nearly 27.1% of deaths were related to liver cancer [3-5]. Although the effect of national HBV vaccination program may possibly diminish the prevalence of HBV infection in the next few years, the incidence and prevalence of HBV-related diseases including LC and HCC were predicted to be gradually increasing for the next two decades due to the long latency of chronic hepatitis B [6].

Chronic HBV infection has been described as a high risk factor of HCC development with a 100-fold increase in chronic HBV carriers compared to non-carriers [5,7,8]. However, other factors including the geographical region, ethnicity, prolonged use of alcohol, environmental factors and hepatitis C virus (HCV) infection also significantly contribute to the HCC incidence [7]. The mechanisms and pathogenesis by which HBV induces HCC still need a detailed investigation. However, several mechanisms were proposed and partly demonstrated including the immune response to infected liver cells during inflammation due to HBV infection, the instability of host chromosome due to the integration of HBV-DNA, modulation and mutation in the coding genes or oncogenes, and the interaction between viral HBx protein with host signaling proteins consequently modulate the hepatocyte proliferation [9,10].

Several therapeutic interventions for HCC including transcatheter oily chemoembolization (TOCE), radiofrequency ablation (RFA), alcohol injection and surgical resection are in place. The choice of treatment mostly depends on the stage of cancer which typically determined based on the Child-Pugh classification system [7]. Additionally, the possible new prognostic biomarkers such as serum and tissue levels of vascular endothelial growth factor (VEGF) and C-C motif chemokine ligand 15 (CCL15) have been recently identified [11,12]. However, the consideration of using these biomarkers in clinical practice is still needed to be investigated.

Many studies have demonstrated the clinical significance of platelets in HBV-related liver diseases including HCC [13-18]. Of which, different mechanisms leading to thrombocytopenia including abnormal platelet production, destruction and platelet-specific glycoproteins levels have been proposed in HBV-related liver disease [13,15]. The blood platelets have been shown *in vitro* to induce growth and invasion of several HCC cell lines [18]. In clinical observations, high blood platelet counts were associated with extrahepatic metastasis [16] and with increased tumor size [17]. In HCC treatment, anti-platelet therapy showed a potential effect on inhibition of HBV-related HCC development in a mouse model [19,20]. Therefore, it would be important to investigate the correlations between platelet counts and clinical parameters during progression of HBV-related liver diseases and response to HCC treatment.

Human major histocompatibility complex (MHC) class I polypeptide-related sequence B (*MICB*) belongs to the *MIC* family located within the MHC class I region of chromosome 6 and consists of two functional genes *MICA* and *MICB* and five pseudogenes *MICC* to *MICG.* The *MICB* gene is 12,930 bp long and contains 6 exons which encoding a protein of 383 amino acids [21]. Similar to MICA, MICB protein interacts with the natural killer cell receptor NKG2D and T cell receptor (TCR) and consequently activates the natural killer (NK) cells, gamma-delta (γδ) T cells and alpha-beta (αβ) CD8+ T cells [22-24]. These activated effector cells (NK, γδT and αβ CD8+ T cells) have been described to modulate immune responses such as anti-tumor, viral infection and autoimmune diseases [21-24]. In particular, NK cells have been shown to play an important role during HBV infection and progression of liver diseases including LC and HCC [25,26]. In addition, MICB protein is also expressed mainly in fibroblasts and epithelial as well as in different cancer cell lines such as cervical cancer and myelomonocytic leukemia [21,27,28]. Also, soluble MICB serum levels were associated with various diseases such as multiple sclerosis, liver diseases and cancers [29-33]. However, to the best of our knowledge there was no study regarding the role of sMICB during HBV infection and the possible association with response to the treatment of HBV-related HCC. In this study, we aim to analyze and validate the prognostic potential of sMICB protein in the progression of HBV-related liver diseases and to treatment response of HBV-related HCC.

## Methods

### Patients

Two hundred and sixty six adult HBV-infected Vietnamese patients were recruited in this study and enrolled at the Tran Hung Dao and 103 Military hospitals of Hanoi, Vietnam during 2012. The study was designed as a cross-sectional study combined with clinical and experimental observations. The patients were classified into five subgroups based on the clinical, biochemical and serological diagnosis and symptomatic patients with histological examination following liver biopsy into those with or without evidence of either cirrhosis or carcinoma. The patient subgroups include asymptomatic HBV carriers (ASYM; n = 48), chronic hepatitis B (CHB; n = 42), liver cirrhosis (LC; n = 49), hepatocellular carcinoma patients without liver cirrhosis (HCC; n = 102) and hepatocellular carcinoma patients with liver cirrhosis (HCC + LC, n = 25). All study subjects were confirmed positive for HBsAg and negative for anti-HCV and anti-HIV. Forty eight healthy Vietnamese blood donors (HC) were included as a control group. The healthy individuals were examined for their healthy status and confirmed negative for HBsAg, anti-HCV and anti-HIV and none of them had a history of alcohol or drug use. All clinical and serological parameters were measured by routine laboratory tests.

### Ethical statement

Informed written consent was received from all studied participants. The study was approved by the Institutional Review Board of the Vietnam Military Medical University (VMMU).

### Classification of hepatitis B patients

The clinical course and severity of hepatitis infections, liver biochemical tests, serological markers of HBV including diagnostic tests for HCC in the chronic HBV infected patients were described as detailed elsewhere [34,35]. Biopsies were taken from all patients with suspected chronic HBV including LC and HCC. The patients were then classified on the basis of detailed histological examination, into those with or without evidence of either cirrhosis or carcinoma. In the latter case, the degree of differentiation was noted. Individuals with neither cirrhosis nor carcinoma were attributed a histological activity index according to the scheme expression on hepatocytes observed by earlier studies [35-37].

### Therapeutic intervention of hepatocellular carcinoma

Of the 127 HCC patients, 76 were treated either with transcatheter oily chemoembolization (TOCE), radiofrequency ablation (RFA), percutaneous ethanol injection (PEI) or surgical resection. Of which, 12 patients were treated with TOCE, RFA and alcohol injection, 23 patients were treated with both TOCE and RFA, 25 patients were treated with both TOCE and alcohol infection, and 15 patients were treated with both RFA and alcohol injection. Six patients who not received any non-surgical therapeutic intervention were intervened by surgical resection (Table 1). In addition, no antiviral therapy was applied for the HCC patients at the time of sampling. The patients were selected for treatment based on tumor size, number of tumor, site of tumor and stage of the liver cancer. The PEI was used for the patients who had liver tumors 3–5 cm and the RFA was used for the patients with 1 tumor < 5 cm, or 3 tumors < 3 cm in size whereas the TOCE was used for the patients who had many tumors and/or big tumors and based on other factors such as the current condition of patients and liver function. Not-treated HCC patients were at later stage of liver cancer, with decompensated liver cirrhosis and/or with metastasis. We collected samples from all of the 76 treated HCC patients one month after the course of therapy has been finished and the patients were not received any medicine. Of the remaining 51 HCC patients, samples were collected at the time of admission to the hospital. No evidence of side effects in treated HCC patients was observed and there was no time window for these treated HCC patients.

**Table 1:** **Characteristics of study subjects segregated according to clinical status**

**Characteristics**	**ASYM (n = 48)**	**CHB (n = 42)**	**LC (n = 49)**	**HCC (n = 102)**	**HCC with LC (n = 25)**
Age (years)	36 [18–57]	39 [20–69]	57 [27–79]	54.5 [26–81]	60 [40–75]
Gender (M/F)	35/13	33/9	41/8	102/0	25/0
WBC* 10^9^/L	6.7 [4.8–11]	5.99 [4.8–11.8]	5.6 [3.2–18]	6 [2.7–17]	5.6 [3–8.4]
RBC* 10^12^/L	5 [3.8–47]	4.8 [3.8–11.6]	4.2 [2.5–5.7]	4.5 [2.43–6]	4 [3–5.2]
PLT* 10^9^/L	227 [117–376]	217 [117–332]	98 [3.7–320]	166 [40–382]	142 [35–226]
ALT* (IU/L)	23 [8–74]	47 [13–750]	57 [13–395]	42 [12–805]	46 [11–290]
AST* (IU/L)	25 [12–35]	38 [19–599]	84 [15–329]	54.5 [17–670]	116 [25–655]
Total bilirubin* (mg/dl)	13 [6.7–32]	16 [8–32]	29 [9–571]	18 [8–184]	27 [9.6–185]
Direct bilirubin* (mg/dl)	3.5 [1–8]	5.7 [1.4–26]	10 [0.4–291]	6 [1–80]	12.5 [2–59]
Albumin* (g/L)	42 [38–48]	43 [12–48]	30 [3.3–47]	38 [4–47]	32 [22–42]
Prothrombin* (% of standard)	92 [78–127]	94 [72–127]	65 [23.4–92]	78 [19.6–128]	69 [36–124]
HBV viral load* (copies/ml)	6.65×10^4^	5.53×10^5^	8.85×10^5^	5.94×10^5^	4.05×10^5^
	[290–6.26×10^9^]	[100–9.9×10^8^]	[203–4.71×10^8^]	[190–2.28×10^9^]	[2040–3.04×10^9^]
Alpha–feto protein (AFP)* (mg/L)	NA	<5	6.8 [1.26–300]	79 [1.38–350]	300 [2.56–300]
Treatments (treated/not-treated)				61/41	15/10
*TOCE*	NA	NA	NA	48/55	11/14
*RFA*	NA	NA	NA	18/85	8/17
*Alcohol injection*	NA	NA	NA	26/77	10/15
*Surgery*	NA	NA	NA	6/97	0/25

ASYM, asymptomatic; CHB, chronic hepatitis B; LC, liver cirrhosis; HCC, hepatocellular carcinoma; WBC, white blood cells; RBC, red blood cells; PLT, platelets; AST and ALT, aspartate and alanine amino transferase; IU, international units; TOCE, transcatheter oily chemoembolization; RFA, radiofrequency ablation; NA, not applicable. Values given are medians and range; (*) *P* < 0.05 for comparison with all other groups.

### Quantification of sMICB serum levels

Soluble MICB serum levels were measured in the sera from the HBV infected patient groups and in healthy controls using a commercially available MICB Human ELISA Kit (Abcam, Tokyo, Japan; Catalog No. ab100593) following the manufacturer’s instructions. Briefly, standard and sample were added into appropriate wells of coated ELISA plate and incubated over night at 4°C with gentle shaking. Plates were washed with wash solution and subsequently biotinylated MICB detection was added to each well and was incubated at room temperature with gentle shaking. After removing the solution and washing the plates, HRP-Streptavidin solution was added to each well and incubated at room temperature with gentle shaking. The plates were washed again and TMB One-Step substrate reagent was added to each well, incubated at room temperature in the dark with gentle shaking. Finally, stop solution was added to each well and plates were read at 450 nm immediately. The standard curve was plotted log-log graph paper based upon the mean of absorbance and the best fit straight line was drawn through the standard points. The concentrations of sMICB serum protein were calculated based upon the standard curve. The minimum detectable limit of sMICB serum proteins was 0.069 ng/ml.

### Statistical analysis

Clinical and demographic data were presented using median with range for continuous variables and the student t tests were used for comparisons of two groups. Kruskal-Wallis or Mann–Whitney U test was used to analyze the sMICB serum levels in patients and in healthy controls and the association of sMICB serum levels with the treatments of HCC where appropriate. The Spearman’s rank correlation coefficient or Pearson product–moment correlation coefficient was used to analyze the correlation of sMICB serum level with clinical and laboratory parameters where appropriate. All statistical analysis was performed using IBM Statistics SPSS v.19 and the level of significance was set at a *P* value of less than 0.05.

## Results

### Clinical characteristics of the studied patients

The main clinical and demographic characteristics such as age, gender, liver biochemical tests, viral load and the tumor marker alpha-feto protein for all the investigated 266 Vietnamese hepatitis B patients and 48 healthy controls are summarized in Table 1. The white blood cells, red blood cells and platelet counts were decreased in CHB, LC and HCC groups compared to the ASYM group. The platelet counts were observed significantly lower in LC and HCC groups compared to ASYM and CHB (*P* < 0.0001). Levels of laboratory parameters such as ALT, AST, total and direct bilirubin and HBV-DNA loads were significantly higher in CHB, LC and HCC groups compared to ASYM group whereas the levels of albumin and prothrombin were significantly decreased in LC and HCC groups compared to ASYM and CHB (*P* < 0.001). As expected, the level of the tumour marker alpha-feto protein was observed significantly higher in HCC patients compared to LC patients (*P* < 0.001). Furthermore, the levels of AST and alpha-feto protein were significantly elevated in HCC patients with liver cirrhosis compared to HCC patients without liver cirrhosis (*P* < 0.001) (Table 1).

### Soluble MICB serum levels in hepatitis B patients

**Figure 1:**
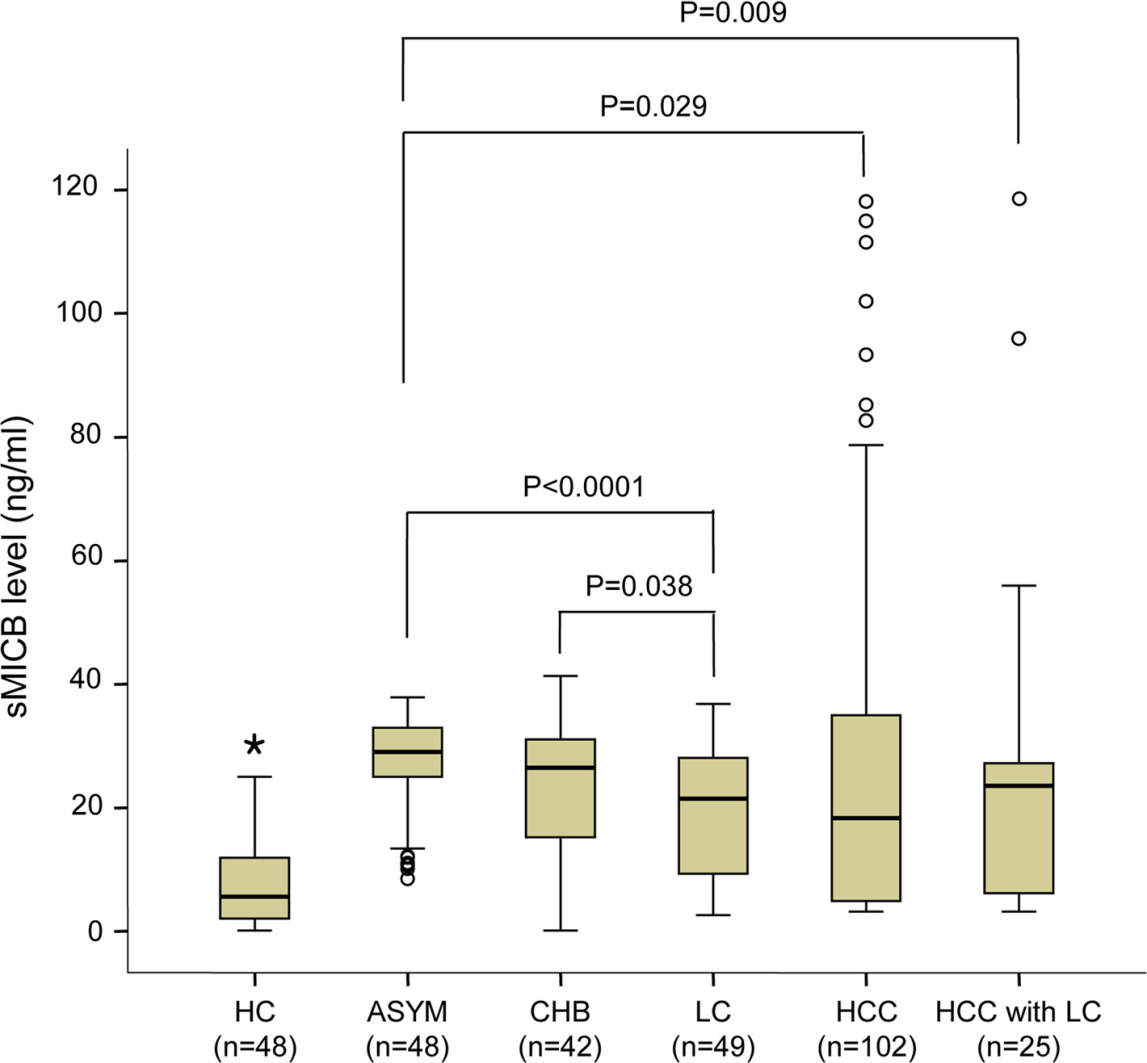
**sMICB serum levels in patient groups and healthy controls.** Soluble MICB serum levels were measured in different subgroups of patient and healthy controls. HC: healthy control, ASYM: asymptomatic, CHB: chronic hepatitis B, LC: liver cirrhosis, HCC: hepatocellular carcinoma. Numbers in parentheses are number of samples measured, (*): *P* < 0.0001 in comparisons of healthy controls with different patient groups using two sided Mann–Whitney U test.

Soluble MICB (sMICB) serum levels were determined in 266 studied HBV patients and 48 healthy controls. We observed a median of 29.07 ng/ml [8.5-37.9] in ASYM, 26.5 ng/ml [0.1-41.3] in CHB, 21.5 ng/ml [2.6-36.8] in LC, 18.35 ng/ml [3.14-195.7] in HCC patients without cirrhosis and 23.6 ng/ml [3.14-118] in HCC patients with cirrhosis. Analyzing healthy controls only a median of 5.6 ng/ml [0.1-25.04] could be detected. The results demonstrated that the sMICB serum levels were significantly elevated in HBV patients compared to healthy controls (*P* < 0.0001) (Figure 1). The sMICB serum levels varied in the patient subgroups, of which, sMICB serum levels were significantly higher in ASYM compared to LC and HCC patient groups (*P* < 0.0001, and *P* = 0.029, respectively). Significantly lower sMICB serum levels were observed in the LC patient group in comparison to the CHB patient group (*P* = 0.038). There was no significant difference of the sMICB serum levels in comparison between ASYM and CHB, CHB and HCC as well as between LC and HCC patient groups (*P* > 0.05). Although compared with HCC patients without liver cirrhosis, patients with liver cirrhosis have a small increase of sMICB level, there was no significance (*P* > 0.05) (Figure 1).

### Relationship between sMICB serum levels and clinical parameters

**Figure 2:**
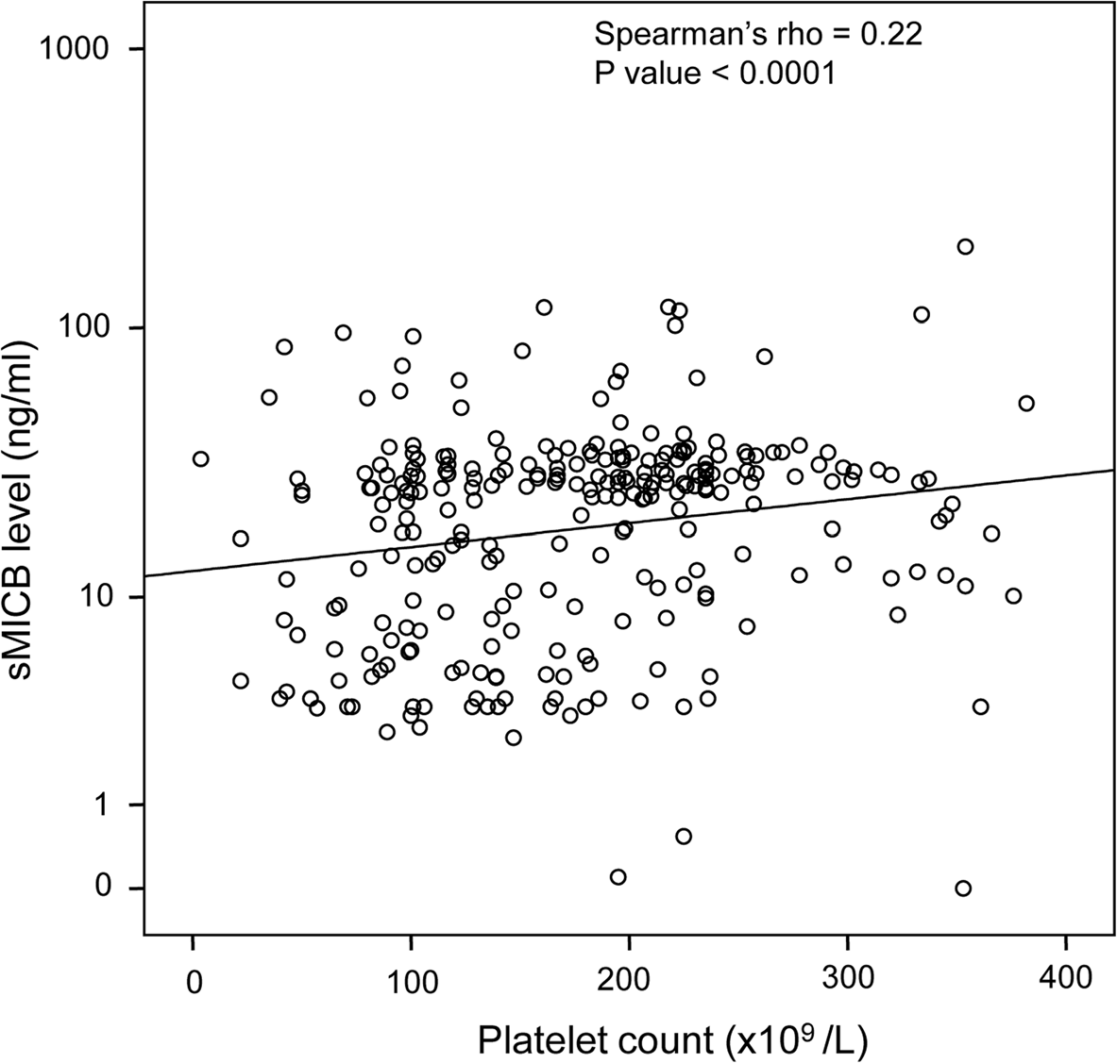
**Correlation between sMICB serum levels and platelet counts in HBV patients.** The sMICB serum levels were correlated with platelet count using Spearman’s rank correlation coefficient test. The Spearman’s rho and *P* value are also presented.

**Figure 3:**
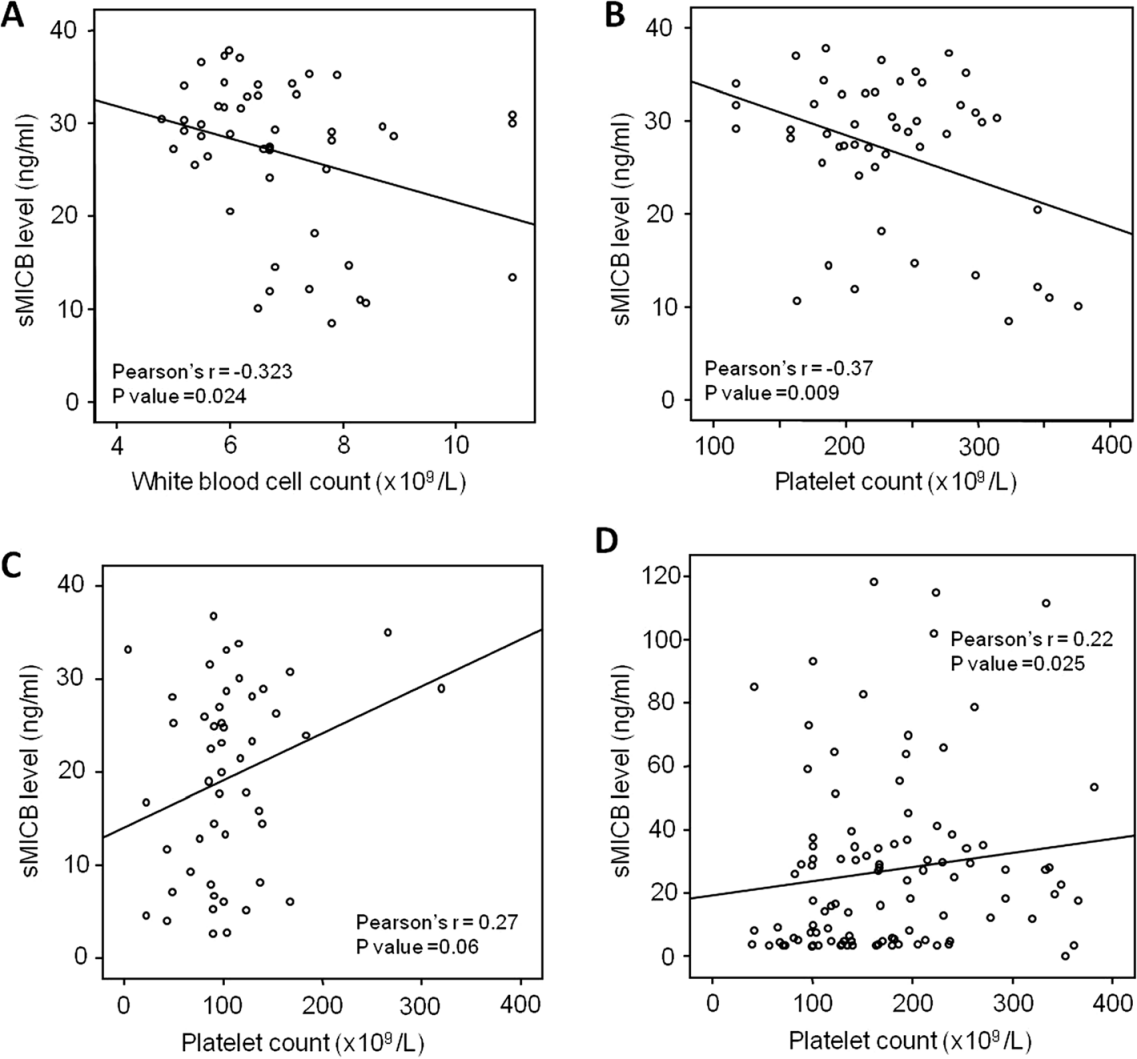
**Correlation between sMICB serum levels and clinical parameters in HBV patient groups.** The sMICB serum levels were correlated with platelet count and white blood cell count using Pearson product–moment correlation coefficient test. The Pearson’s r and *P* values are also presented. **(A)**: correlation between sMICB serum levels and white blood cell count in asymptomatic HBV carriers, **(B)**: correlation between sMICB serum levels and platelet in asymptomatic HBV carriers, **(C)**: correlation between sMICB serum levels and platelet in liver cirrhosis patients, **(D)**: correlation between sMICB serum levels and platelet in hepatocellular carcinoma patients.

In order to analyze the relationship between sMICB serum levels and clinical parameters, the sMICB serum levels were correlated with all available clinical and laboratory parameters in the different HBV-infected patient groups. In pooled HBV patients, we notably observed a significantly positive correlation between sMICB serum levels and platelet counts (Spearman’s rho = 0.22, *P* < 0.0001) (Figure 2A). However, the sMICB serum levels were not correlated with white and red blood cell counts, ALT, AST, total and direct bilirubin, albumin, prothrombin, AFP and HBV-DNA viral loads. When patients were divided into clinical subgroups, we observed that the sMICB serum levels were significantly reverse correlated with white blood cell counts and platelet counts in ASYM group (Pearson’s r = −0.323, *P* = 0.024 and Pearson’s r = −0.37, *P* = 0.009, respectively) (Figure 3A and B). We also observed a positive correlation between sMICB serum levels and platelet counts in LC and HCC groups (Pearson’s r = 0.27, *P* = 0.06 and Pearson’s r = 0.22, *P* = 0.025, respectively) (Figure 3C and D). However, no significant correlations were observed between sMICB serum levels and several available clinical and laboratory parameters in CHB patient group (data not shown).

### Soluble MICB serum levels and hepatocellular carcinoma treatment

**Figure 4:**
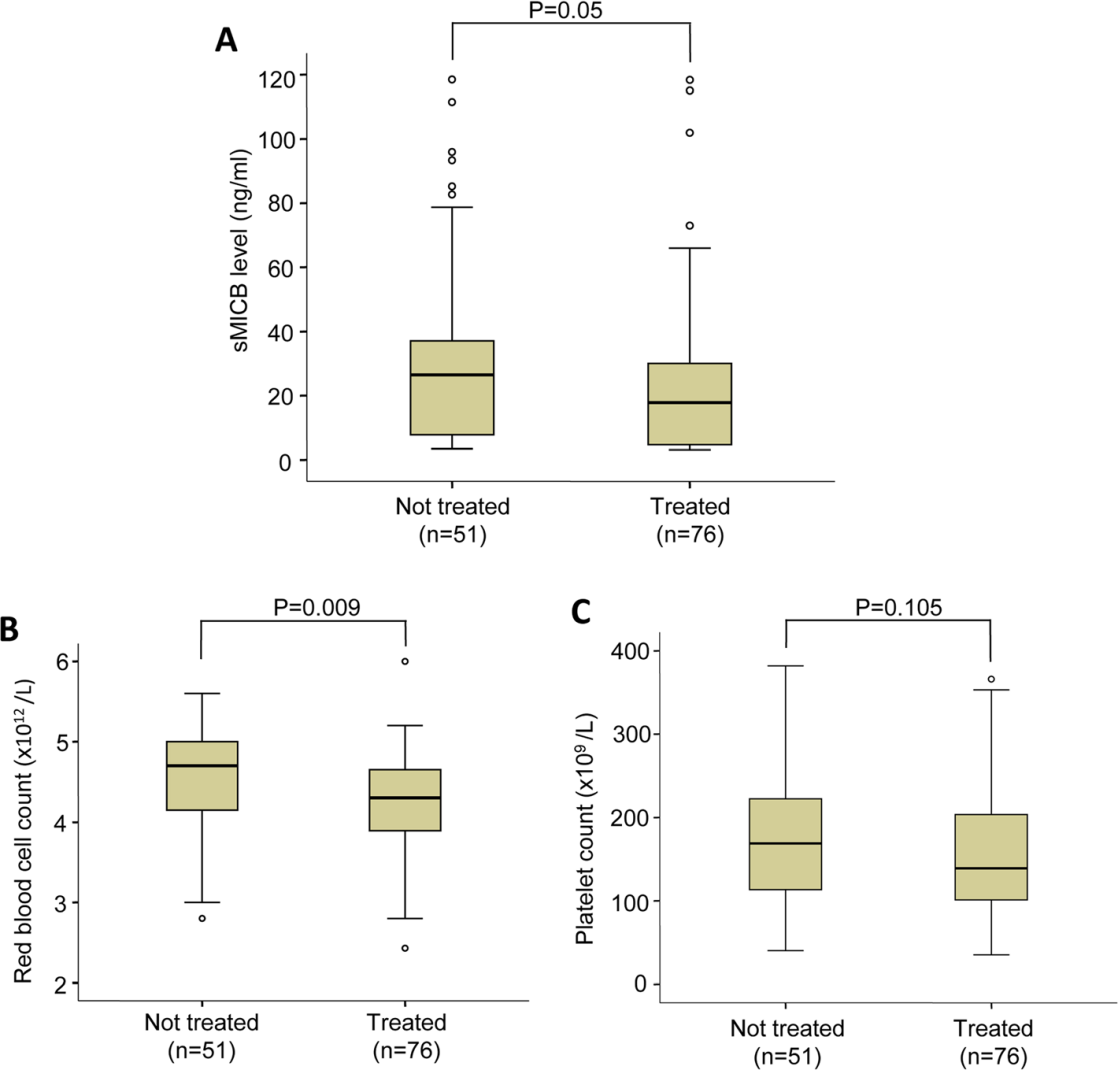
**sMICB serum levels and clinical parameters according to HCC treatment. (A)**: The sMICB serum levels in not-treated and treated HCC patients. **(B)**: Red blood cell counts in not-treated and treated HCC patients. **(C)**: Platelet count in not-treated and treated HCC patients. *P* values were calculated by using two sided Mann–Whitney U test.

Of the 127 studied HCC patients, 76 HCC patients were treated with different cancer therapies including transcatheter oily chemoembolization (TOCE), radiofrequency ablation (RFA), alcohol injection and surgical resection. The remaining 51 HCC patients were yet to be treated with any cancer therapies. Analysis of the sMICB serum levels in treated versus not-treated HCC patient groups, we observed a decreased sMICB serum level in treated HCC patient group compared to not-treated HCC patient group (*P* = 0.05) (Figure 4A). However, there were no significant differences of sMICB serum levels in different modalities of therapeutic interventions (data not shown). In contrast, the red blood cell counts were significantly decreased in treated HCC patient group compared to not-treated HCC patient group (*P* = 0.009). The platelet counts decreased but with no statistically significance in treated HCC patients compared with not-treated HCC patients (*P* = 0.105) (Figure 4B and C).

## Discussion

Human major histocompatibility complex (MHC) class I polypeptide-related sequence B (MICB), together with MICA are ligands of the NKG2D receptor. The interaction between NKG2D receptor and its ligand results in an activation of NK cells, γδ T cells and αβ CD8+ T cells which play a crucial role during viral infection, autoimmune disease and tumor surveillance [21-24]. In this study, we investigated the levels of sMICB serum protein in different stages of HBV infection and its role in HCC treatment. Our results showed that the sMICB serum levels were significantly increased in HBV patients compared to healthy controls (*P* < 0.0001), significantly decreased in treated HCC patients compared to not-treated HCC patients (*P* = 0.05), and significantly correlated with platelet count in ASYM, LC and HCC patients. These findings propose that sMICB serum protein significantly contribute to in immune response to the HBV infection, plays a role in progression of liver diseases and is possibly considered as biomarker for response to HCC treatment.

The increased sMICB serum protein has been previously demonstrated to correlate with different cancers compared to healthy individuals [29-33]. Several studies revealed that elevated sMICB serum levels were correlated with stages of tumor and metastasis as well as in the progression of both HBV and HCC related liver diseases [29,30]. In contrast, the sMICB serum levels in this study were not elevated with the progression of liver diseases. The contrary results could be explained by the cellular stress response mechanisms during HBV infection by which the promoter heat shock elements regulate the expression of MICA and MICB similar to those of HSP70 [38]. This hypothesis is supported by several studies which demonstrate that MICB was not only expressed in tumor cells but also in different non-tumor cell lines under stress conditions [27,28,39,40]. Therefore, due to viral infections and long-term inflammation, MIC proteins (MICA and MICB) can be over-expressed. In addition, the higher sMICB serum levels were observed in asymptomatic HBV carriers that are consistent with several previous studies which reported the elevated sMICA serum levels in early stage of viral infection [41,42] and inferred that MICA expression was induced by viral infection through cellular stress response mechanisms [42]. A very recent study has shown that over-expression of HBsAg suppresses the expression of MICA and MICB via induction of several cellular miRNAs [43]. Therefore, lower levels of sMICB protein in the patients with chronic hepatitis B, LC and HCC could be best explained by the high levels of HBsAg due to the higher level of HBV replication in the later stage of HBV infection such as LC and HCC. A limitation of our study is that the information for antiviral therapy of the CHB and LC patients was not completed therefore we could not analyze the effect of antiviral therapy on sMICB level. Nevertheless, we assume that the antiviral therapies possibly influence the sMICB level and further studies are needed to verify the hypothesis.

Concerning the role of sMICB in HCC treatment, our results showed that sMICB serum levels were decreased in treated HCC patients compared to untreated HCC patients but were not associated with different modalities of HCC treatment. Conversely, earlier study showed that the sMICB serum levels were strongly increased in melanoma patients treated with cytostatics compared to untreated patients. However, other therapy such as IFN-α treatment did not affect the sMICB serum levels [33]. In addition, another study showed that there was no significant difference of sMICB serum levels between multiple sclerosis patients with or without immunomodulatory treatment [44]. Hence, different therapy modalities might inversely affect the sMIC (sMICA and sMICB) serum levels. In case of HCC, a study has indicated that the sMICA serum levels, but not sMICB serum levels, were significantly decreased after transcatheter arterial embolization (TAE) therapy. However, when analyzing the subpopulation of the HCC patient group in our study, sMICB serum levels were decreased in HCC patients with compensated cirrhosis in contrast to HCC patients with mildly decompensated cirrhosis and severely decompensated cirrhosis [30]. Due to the important roles of MIC proteins in modulation of immune response, a study has proposed that serum tests of sMICA and sMICB in combination with tumor measurements could be considered to enhance the effectiveness of immune therapeutic interventions [45]. From our results, we suggest that sMICB and sMICA serum levels [30,42,46] could serve as a biomarker for monitoring treatment outcome of HBV-induced HCC.

A recent study using a mouse model of chronic immune-mediated hepatitis B has indicated that anti-platelet drugs effectively prevent HCC and improve survival suggesting that platelets could play a crucial role in the pathogenesis of HBV-associated liver cancer [19,20]. In agreement with this study, our results indicated that the platelet counts were significantly decreased according to the progression of liver disease. Interestingly, we observed a positive correlation between sMICB serum levels and platelet counts in studied HBV infected patients and in patient subgroups including LC and HCC whereas an inverse correlation was observed in ASYM carriers. An explanation for that finding could be a strong decrease of platelet counts in the later stage of liver disease like LC and HCC while the sMICB serum levels were not decreased or even increased during HCC pathogenesis. However, the platelet counts were not different between HCC patients with and without treatment indicating that the therapeutic interventions employed in our study did not affect the platelet counts. The platelets have been proposed to be recruited into the liver and facilitate the hepatic accumulation of virus specific CD8+ T cells which immune-mediated liver injury during HBV infection [20,47]. Additionally, platelets were also implied to be involved in accumulation of virus-nonspecific CD8+ T cells and other virus-nonspecific inflammatory cells including NK cells during acute hepatitis in a mouse model [19,20,47]. Recent reports have shown that the platelet counts were inverse correlated with mean platelet volume and suggested that mean platelet volume could determine the severity of liver fibrosis and inflammation in CHB patients [48,49]. The observation of lowest platelet counts in the LC group in our study compared to other HBV patient groups may support earlier findings that platelets could be a crucial player during progression of LC and HCC [19,20]. In addition, post-operative low platelet counts have been shown to be associated with poor outcome after hepatic resection for HCC suggesting an important role of platelets during liver compensation [50]. However, further detailed studies are needed to detail the clinical role of platelets during liver disease and immune response in HCC treatment.

## Conclusion

Our results showed elevated sMICB serum levels in different stages of liver disease progression compared to healthy controls, and decreased sMICB serum levels in treated HCC patients compared to untreated HCC patients. Notably, sMICB serum levels were significantly correlated with platelet counts which were speculated recently as a key player during development of liver cirrhosis and hepatocellular carcinoma therapy. Our findings suggest that sMICB could play a vital role during HBV infection, liver disease progression and HCC treatment.
